# Prognostic value of eight immune gene signatures in pancreatic cancer patients

**DOI:** 10.1186/s12920-020-00868-w

**Published:** 2021-02-05

**Authors:** Wenting Wang, Zhijian Xu, Ning Wang, Ruyong Yao, Tao Qin, Hao Lin, Lu Yue

**Affiliations:** 1 Qingdao Municipal Hospital, School of Medicine, Qingdao University, 5 Donghaizhong Road, Qingdao, 266071 Shandong People’s Republic of China; 2 Department of Central Laboratory, The Affiliated Hospital of Qingdao University, 16 Jiangsu Road, Qingdao, 266003 Shandong People’s Republic of China

## Abstract

**Background:**

Pancreatic cancer is one of the most common malignant tumors of the digestive tract, and it has a poor prognosis. Traditional methods are not effective to accurately assess the prognosis of patients with pancreatic cancer. Immunotherapy is a new promising approach for the treatment of pancreatic cancer; however, some patients do not respond well to immunotherapy, which may be related to tumor microenvironment regulation. In this study, we use gene expression database to mine important immune genes and establish a prognostic prediction model for pancreatic cancer patients. We hope to provide a feasible method to evaluate the prognosis of pancreatic cancer and provide valuable targets for pancreatic cancer immunotherapy.

**Results:**

We used univariate COX proportional hazard regression analysis, the least absolute shrinkage and selection operator, and multivariate COX regression analysis to screen 8 genes related to prognosis from the 314 immune-related genes, and used them to construct a new clinical prediction model in the TCGA pancreatic cancer cohort. Subsequently, we evaluated the prognostic value of the model. The Kaplan–Meier cumulative curve showed that patients with low risk scores survived significantly longer than patients with high risk scores. The area under the ROC curve (AUC value) of the risk score was 0.755. The univariate COX analysis showed that the risk score was significantly related to overall survival (HR 1.406, 95% CI 1.237–1.598, P < 0.001), and multivariate analysis showed that the risk score was an independent prognostic factor (HR 1.400, 95% CI 1.287–1.522, P < 0.001). Correlation analysis found that immune genes are closely related to tumor immune microenvironment.

**Conclusions:**

Based on the TCGA-PAAD cohort, we identified immune-related markers with independent prognostic significance, validated, and analyzed their biological functions, to provide a feasible method for the prognosis of pancreatic cancer and provide potentially valuable targets for pancreatic cancer immunotherapy.

## Background

Pancreatic cancer is a common malignant tumor of the digestive tract, which has the characteristics of difficult early diagnosis, rapid metastasis, and poor therapeutic effect. It is one of the most invasive and fatal malignant tumors in the world, and the prognosis is very poor. The five-year survival rate is less than 8% [1]. Surgery is the most important and comprehensive treatment for pancreatic cancer, and most patients have recurrence and metastasis earlier after operation. It is difficult to determine the overall postoperative annual survival rate. Postoperative adjuvant radiotherapy and chemotherapy aim to kill residual tumor cells to improve the curative effect; however, due to the difficulties of early diagnosis and the lack of effective adjuvant treatment, the effect is very little [2, 3]. Up to now, the prediction and prognosis of pancreatic cancer mainly depend on histopathological diagnosis and tumor staging system. However, traditional methods are not enough to accurately evaluate the prognosis of patients with pancreatic cancer and cannot meet the needs of clinicians to assist in diagnosis and treatment [4]. Therefore, it is imperative to develop reliable and accurate prognostic biomarkers to help clinicians optimize treatment strategies.

With the increased understanding on the key role of immune system in the development of pancreatic cancer, immunotherapy such as chimeric antigen receptor T cell therapy and immune checkpoint inhibitor (ICBs) is very promising for the treatment of pancreatic cancer patients. Exciting results have been achieved in preclinical and clinical trials of pancreatic cancer [5, 6]. However, according to the latest research, the efficacy of immunotherapy is affected by many factors, such as tumor microenvironment, microsatellite instability, and tumor mutation load. In addition, some patients develop drug resistance after a period of remission, and even the vast majority of patients are not sensitive to ICB [7]. To develop better strategies to enhance immunity, researchers have conducted several in-depth studies on the matrix response of pancreatic cancer and its interaction with the immune microenvironment of pancreatic cancer. Riquelme et al. found that the development of pancreatic cancer in the mouse model is related to the enrichment of specific strains of bacteria in the intestinal tract and tumor. These strains induce tolerance and immunosuppressive microenvironment, which is conducive to the progression of cancer and resistance to immunotherapy. Eliminating microorganisms with antibiotics can reshape the tumor microenvironment, induce T cell activation, improve immune monitoring, and improve the sensitivity of immunotherapy to established tumors [8]. Loss or inhibition of CXCR2 can improve T cell entry, and combined inhibition of CXCR2 and PD1 in mice with confirmed disease can significantly prolong survival and inhibit pancreatic tumorigenesis and pancreatic cancer metastasis [9]. Winograd et al. found that in pancreatic cancer (a non-immunogenic tumor), the baseline refractory of checkpoint inhibitors can be saved by inducing T cell response with α-CD40/ chemotherapy [10]. These studies have shown that the efficacy of immunotherapy for pancreatic cancer can be significantly improved by changing the tumor immune microenvironment, such as increasing the density of intratumoral effector T cells or inhibiting immunosuppressive cells and receptors. However, the immune microenvironment of pancreatic cancer and its role in the immune escape of cancer cells still need to be understood. It is also urgent to find more new targets to regulate the immune microenvironment to improve the efficacy of immune checkpoint therapy [11].

In recent years, gene expression database has been used to mine valuable therapeutic genes, identify promising prognostic factors, and analyze the molecular mechanisms of various cancers. We used univariate Cox proportional hazard regression analysis, the least absolute shrinkage and selection operator (LASSO), and multivariate COX regression analysis to screen 8 genes related to prognosis from the 314 immune-related genes in the TCGA pancreatic cancer cohort. These can be used as the potential prognostic indicators of pancreatic cancer. The genes were used to establish the optimal risk model; survival analysis, univariate Cox proportional hazard regression analysis, and multivariate Cox proportional hazard regression analysis were used to evaluate the prognostic value of risk score. ROC curve and principal component analysis (PCA) were used to evaluate the accuracy of the model. Patients were divided into high-risk and low-risk groups according to the median risk score. Gene ontology (GO), Kyoto gene and genome encyclopedia (KEGG), and gene set enrichment analysis (GSEA) were used to explore the differences of key signal pathways between high-risk and low-risk groups. Single sample gene set enrichment analysis (ssGSEA) method was used to quantify immune cell infiltration, and the relationship between immune risk genes and tumor immune microenvironment was analyzed. The objectives of this study are to provide a feasible method to evaluate the prognosis of pancreatic cancer, to provide a powerful means of tumor prevention and treatment for regulating the body’s immunity against tumor, and to add new content in the development of new adjuvant drugs targeted at tumor immunotherapy.

## Methods

### Data collection

IMMUNE_RESPONSE and IMMUNE_SYSTEM_PROCESS2 immune gene sets were obtained from Molecular Signatures Database (MSigDB), and a total of 314 immune-related genes were obtained. mRNA expression data and clinical data of 183 pancreatic cancer samples were obtained from the Cancer Genome Atlas (TCGA) database(https://portal.gdc.cancer.gov/).

### Construction of prognostic signature for TCGA pancreatic cancer cohorts

Univariate Cox regression analysis of 314 immune-related genes was carried out to analyze the genes significantly related to the overall survival (OS) of pancreatic cancer. Then LASSO regression analysis was performed on these immune genes. LASSO regression analysis reduced the dimensionality of high-dimensional data by the sum of the absolute values of the limiting coefficients less than the predetermined value, and the variables with relatively small contributions will be given zero coefficients. Only the genes with non-zero coefficient in LASSO regression analysis were selected for further multivariate Cox regression analysis, and the resulting genes were used to build a predictive model. The risk score of each patient was calculated according to the mRNA expression level and risk coefficient of each risk gene, which was calculated by the following equation:

$$\begin{aligned} {\text{risk score}} & = {\text{Expression}}_{{{\text{mRNA1}}}} \times {\text{Coefficient}}_{{{\text{mRNA1}}}} + {\text{Expression}}_{{{\text{mRNA2}}}} \\ & \quad \times {\text{Coefficient}}_{{{\text{mRNA2}}}} + \cdots {\text{Expression}}_{{{\text{mRNAn}}}} \times {\text{Coefficient}}_{{{\text{mRNAn}}}} \\ \end{aligned}$$

Taking the median risk score as the cut-off value, the patients were divided into high-risk and low-risk groups for follow-up analysis.

### Pathway analysis

The R language “EdgeR” package calculation was used to analyze the difference of mRNA between low-risk and high-risk groups. mRNA with FDR value less than 0.05 was annotated by Gene Ontology (GO, http://geneontology.org/), and the biological functions of different genes, including biological processes, cellular components, and molecular functions, were analyzed. The Kyoto Encyclopedia of genes and Genomes (KEGG, https://www.genome.jp/kegg/) analyzes a variety of biological information, including metabolic pathways, predicts the function of genes, and analyzes the roles of proteins and other macromolecules. The metabolic pathways and signal transduction pathways of significant enrichment in pancreatic cancer were revealed by pathway enrichment analysis (FDR < 0.05). Then, we performed Gene Set Enrichment Analysis (GSEA, http://software.Broadstitute.org/GSEA/) to reveal the signal pathways and biological processes in which differentially expressed genes were enriched between high-risk and low-risk subpopulations. The "ClusterProfiler" R package was used for visualization [12].

### Calculate the composition of tumor immune microenvironment

To explore the role of immune genes and tumor immune microenvironment, we quantified the level of immune cell infiltration in TCGA pancreatic cancer cohort(TCGA-PAAD) samples. According to the immune cell marker genes, provided by Bindea et al. [13], we used R language "GSVA" package according to the expression of immune cell marker genes in TCGA-PAAD. Single-sample gene set enrichment analysis (ssGSEA) was used to quantify the infiltration level of 24 types of immune cells in the sample, such as T lymphocytes, dendritic cells, and natural killer cells [14]. Pearson's method was used to calculate the correlation between risk genes and immune cells. The TIMER database was used for verification [15].

### Statistical analysis

All statistical analyses were carried out by R programming language (https://www.r-project.org/). R language "Survival" package and "survminer" package were used to draw Kaplan–Meier curve. Univariate and multivariate Cox proportional hazard regression analyses were also used to evaluate the relationship between risk scores and OS. ROC analysis was used to examine the sensitivity and specificity of survival prediction using the gene signature risk score. An area under the ROC curve (AUC) served as an indicator of prognostic accuracy. A P-value of less than 0.05 was set as statistically significant for all the analyses.

## Results

### Acquisition of immune risk genes

**Fig. 1:**
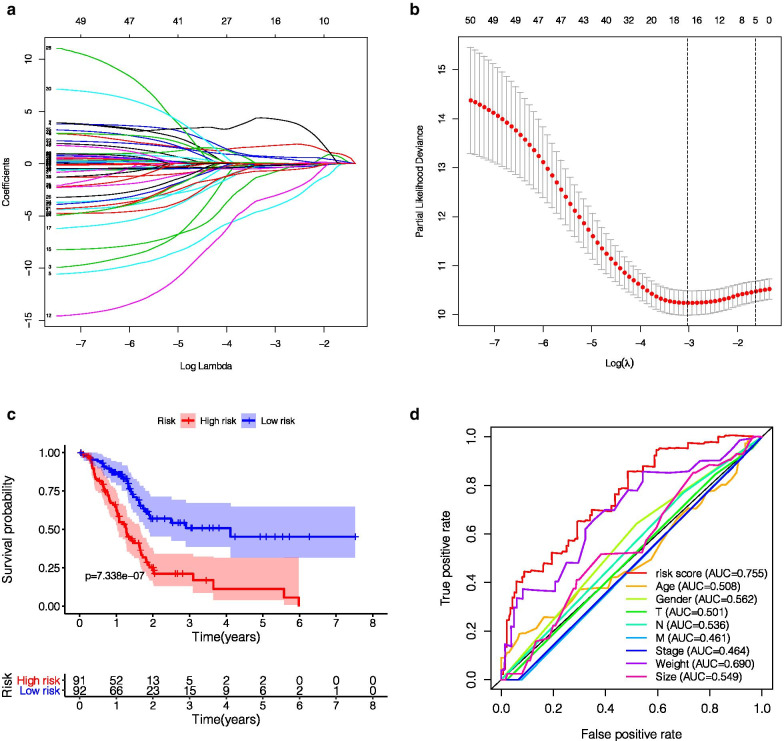
The establishment and verification of the model **a** LASSO coefficient profiles of the 109 immune-genes in TCGA-PAAD. **b** A coefficient profile plot was generated against the log (lambda) sequence. **c** Kaplan–Meier analysis of TCGA-PAAD patients stratified by the median risk score. **d** The sensitivity and specificity of the ROC curve were used to evaluate the model

**Fig. 2:**
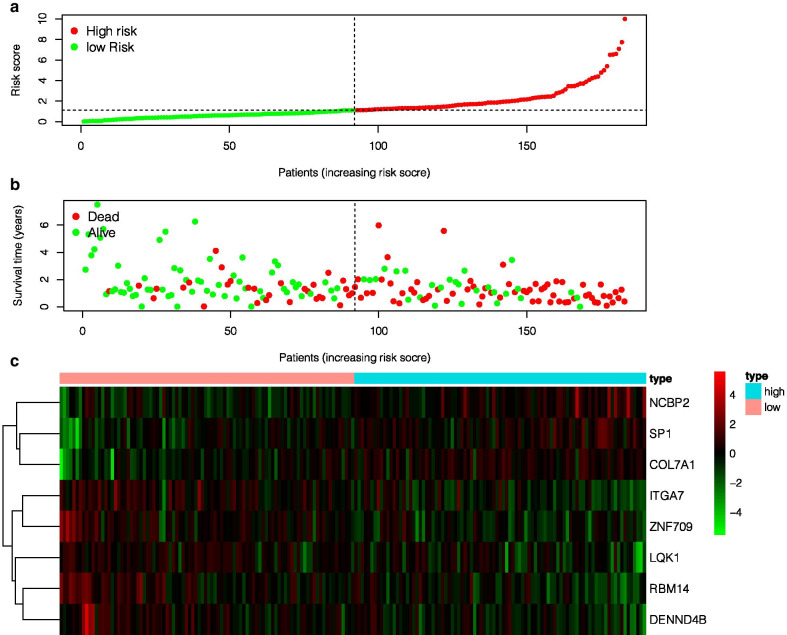
**a** The black dotted line is the best dividing line for dividing patients into low-risk and high-risk groups. **b** The distribution of survival status and survival time of patients with TCGA-PAAD. **c** Heatmap of immune gene expression profile in prognostic markers

**Fig. 3:**
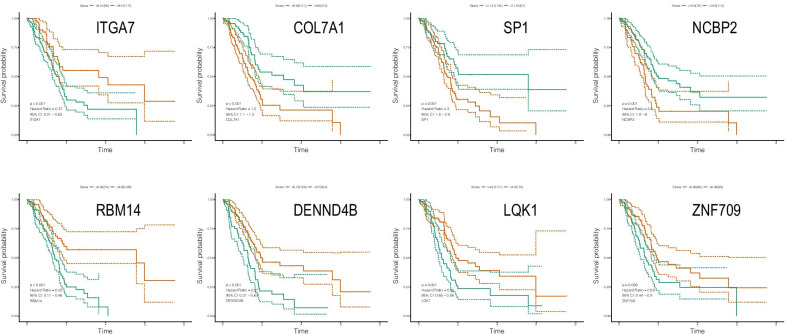
Kaplan–Meier analysis of the effects of 8 immune genes on overall survival in TCGA-PAAD patients

A total of 314 immune-related genes were collected from MSigDB. The expression level and prognosis data of PAAD-related genes were obtained from the TCGA database. Univariate Cox regression analysis was carried out. A total of 109 immune genes with P < 0.05 were selected for lasso regression analysis. When 16 immune gene models were obtained according to the lambda.min value, the performance of 16 immune gene model was the best (Fig. 1b). The 16 genes were analyzed by multivariate COX regression analysis. A total of 8 immune genes (ITGA7, RBM14, DENND4B, LQK1, ZNF709, COL7A1, SP1, NCBP2) were identified as independent prognostic factors of pancreatic cancer, which were used to construct a clinical predictive model (as shown in Table 1). The expression profiles of these genes in high- and low-risk groups are shown by heat map (Fig. 2c), and the Kaplan–Meier curves of these genes are drawn by R language "Survival" package and "survminer" package. The survival time of patients with high expression of ITGA7, RBM14, DENND4B, LQK1, and ZNF709 was significantly longer than that of the patients with low expression, and the prognosis of patients with high expression of COL7A1, SP1, and NCBP2 was worse (Fig. 3).

**Table 1:** Univariate Cox regression analysis and multivariate COX regression analysis results of 8 immune genes

Gene_symbol	Ensembl_ID	Univariate Cox regression				Multivariate Cox regression			
		HR	95% CI lower	95% CI upper	P value	HR	95% CI lower	95% CI upper	P value
ITGA7	ENSG00000135424.15	0.706	0.578	0.863	0.001	0.844	0.681	1.047	0.124
COL7A1	ENSG00000114270.15	1.202	1.079	1.340	0.001	1.172	1.030	1.334	0.016
SP1	ENSG00000185591.9	2.965	1.800	4.884	< 0.001	1.6761	0.951	2.955	0.074
NCBP2	ENSG00000114503.10	3.309	1.810	6.049	< 0.001	2.185	1.079	4.424	0.030
RBM14	ENSG00000239306.4	0.238	0.117	0.483	< 0.001	0.392	0.171	0.899	0.027
DENND4B	ENSG00000198837.9	0.367	0.212	0.636	< 0.001	0.520	0.280	0.967	0.039
LQK1	ENST00000356684.8	0.757	0.653	0.877	< 0.001	0.844	0.722	0.985	0.032
ZNF709	ENSG00000242852.6	0.592	0.436	0.802	0.001	0.732	0.482	1.113	0.144

### Construction and verification of prognostic signature for TCGA-PAAD cohorts

**Fig. 4:**
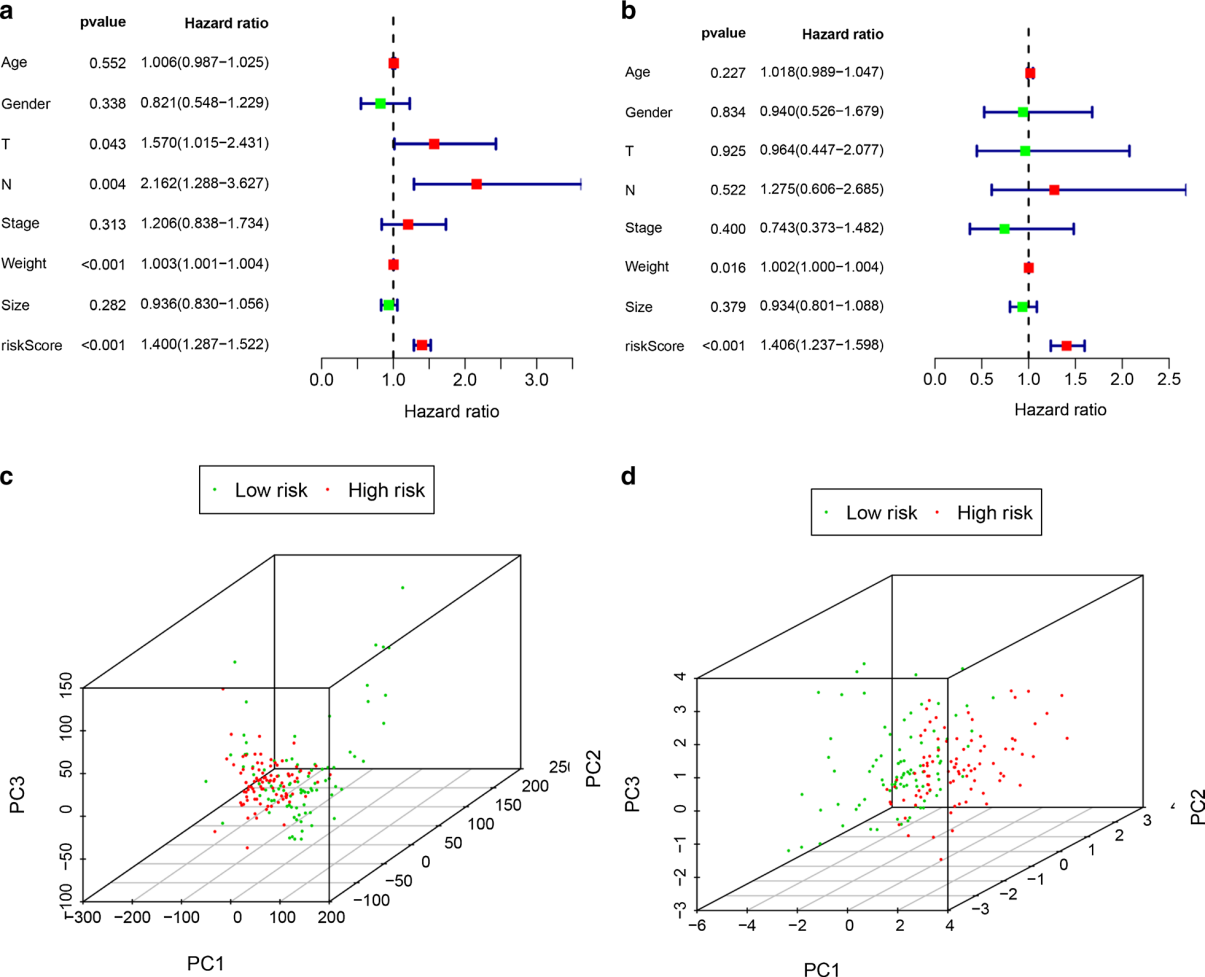
Univariate Cox proportional hazard regression analysis (**a**) and multivariate Cox proportional hazard regression analysis (**b**) explored the correlation between risk score, age, sex, grade, T, N, M, smoking and OS. **c** The expression patterns of grouped samples were analyzed by PCA using all mRNAs. **d** The expression patterns of grouped samples were analyzed by PCA using Prognostic signature

The risk score of each patient was calculated according to the mRNA expression level of each risk gene and the risk coefficient. With the median risk score as the cut-off value, patients were divided into high- and low-risk groups. Kaplan–Meier accumulation curve showed that patients with low-risk score survived significantly longer than those with high-risk score (Fig. 1c). In the TCGA-PAAD cohort, the area under the curve (AUC value) of the risk score was 0.755 (Fig. 1d). Subsequently, we evaluated the prognostic value of the risk score, and univariate COX analysis showed that the TCGA-PAAD risk score was significantly correlated with the overall survival (OS) (HR 1.406, 95%CI 1.237–1.598, P < 0.001) (Fig. 4a). Multifactorial analysis showed that the risk score was an independent predictive factor (HR 1.400, 95%CI 1.287–1.522, P < 0.001) (Fig. 4b). Finally, based on the whole genome expression set, total immunity gene set, and immune risk gene set, we used principal component analysis (PCA) to study the different distribution patterns between low- and high-risk population. When PCA was performed based on the whole genome expression profile, there was no significant segregation in immune status in each group (Fig. 4c). According to the immune risk gene set, low-risk group and high-risk group tended to be divided into two groups (Fig. 4d). The risk score distribution of PAAD patients and the relationship between risk score and survival time are shown in Fig. 2. With the increase of risk value, the number of deaths increases significantly, and the survival time of low-risk group is significantly higher than that of the high-risk group.

### Signaling pathway analysis

**Fig. 5:**
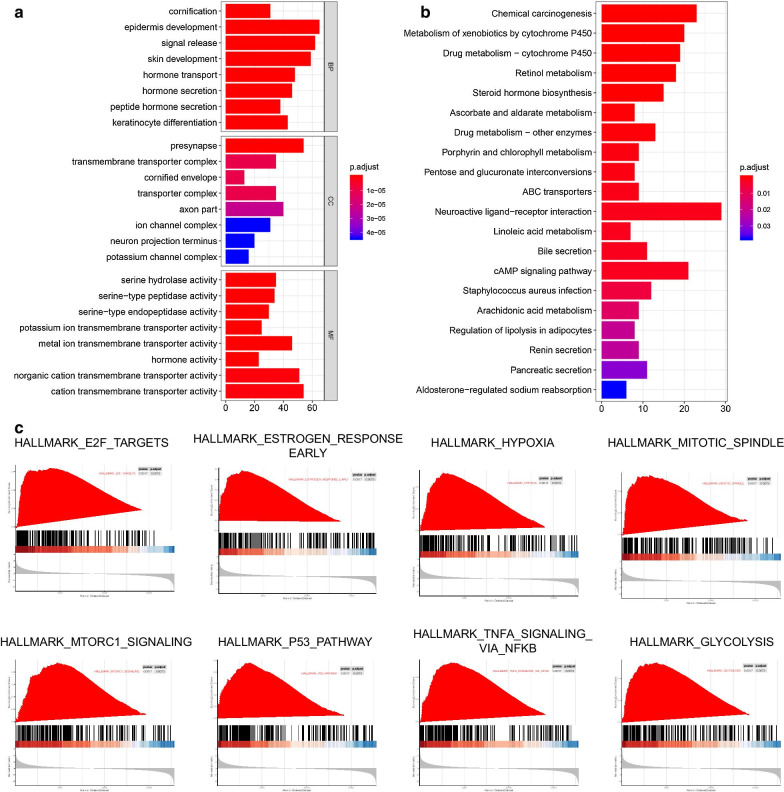
Signaling pathway analysis **a** GO signal pathway enrichment analysis of differential mRNA between low-risk group and high-risk group **b** KEGG signal pathway enrichment analysis was used to analyze the difference of mRNA between low-risk group and high-risk group. **c** GSEA signal pathway enrichment analysis was used to analyze the difference of mRNA between low-risk group and high-risk group

To explore the potential signal pathways related to the immune risk genes, we used edgeR package calculation to analyze the difference of mRNA between low-risk group and high-risk group and also to analyze the signal pathway of mRNA whose FDR value was less than 0.05. GO analysis showed that these genes could be classified into several basic biological processes, including biological development, hormone secretion, synthesis of transmembrane transporter complex, and important transmembrane transporter activity (Fig. 5a). KEGG analysis showed that these genes mainly interact with chemical carcinogenic, neuroactive ligand receptor interaction and cAMP signaling pathways (Fig. 5b, Table 2). By calculating the multiplication of mRNA expression levels of all protein-coding genes between high-risk group and low-risk group, and using GSEA analysis, it was found that the altered genes were significantly enriched in several common pathways. The high-risk group was positively correlated with epithelial-mesenchymal transformation, glycolysis, MTORC1 signal pathway, p53 channel, hypoxia, apoptosis, E2F targets, MYC targets v1, MYC targets v2, TNFA Signaling via NFKB, and inflammatory response, while the low-risk group was positively correlated with pancreas beta cells, allograft rejection and bile acid metabolism (Fig. 5c, Table 3).

**Table 2:** KEGG analysis of the main enriched signal pathways of differential genes between low-risk group and high-risk group

ID	Description	Gene ratio	Bg ratio	P value	P adjust	q value
hsa00830	Retinol metabolism	15/303	67/6589	2.43E−07	3.65E−05	3.02E−05
hsa04080	Neuroactive ligand-receptor interaction	36/303	340/6589	1.79E−06	0.000134	0.000111
hsa04512	ECM-receptor interaction	15/303	88/6589	9.28E−06	0.000464	0.000384
hsa00982	Drug metabolism—cytochrome P450	12/303	72/6589	9.22E−05	0.003457	0.002862
hsa04024	cAMP signaling pathway	22/303	216/6589	0.000351	0.010534	0.008723
hsa04610	Complement and coagulation cascades	12/303	85/6589	0.000462	0.011548	0.009563
hsa04640	Hematopoietic cell lineage	13/303	99/6589	0.000554	0.011874	0.009832
hsa00040	Pentose and glucuronate interconversions	7/303	34/6589	0.000746	0.012988	0.010755
hsa00360	Phenylalanine metabolism	5/303	17/6589	0.000779	0.012988	0.010755
hsa00350	Tyrosine metabolism	7/303	36/6589	0.00107	0.016002	0.013251
hsa00053	Ascorbate and aldarate metabolism	6/303	27/6589	0.001173	0.016002	0.013251
hsa04260	Cardiac muscle contraction	11/303	86/6589	0.001815	0.022681	0.018782
hsa00590	Arachidonic acid metabolism	9/303	63/6589	0.00216	0.024919	0.020635
hsa00980	Metabolism of xenobiotics by cytochrome P450	10/303	76/6589	0.002343	0.025099	0.020784
hsa00860	Porphyrin and chlorophyll metabolism	7/303	42/6589	0.002731	0.027309	0.022614
hsa00983	Drug metabolism—other enzymes	10/303	79/6589	0.003131	0.029357	0.024309
hsa00500	Starch and sucrose metabolism	6/303	36/6589	0.005453	0.048118	0.039845

**Table 3:** GSEA analysis of main enrichment pathways of differential genes between low-risk group and high-risk group

Description	Set size	Enrichment score	NES	P value	P adjust	q values
HALLMARK_ESTROGEN_RESPONSE_LATE	196	0.480174	1.796374	0.001675	0.007284	0.003834
HALLMARK_MITOTIC_SPINDLE	196	0.54578	2.04181	0.001675	0.007284	0.003834
HALLMARK_EPITHELIAL_MESENCHYMAL_TRANSITION	194	0.595047	2.218997	0.001678	0.007284	0.003834
HALLMARK_GLYCOLYSIS	197	0.54494	2.038302	0.001678	0.007284	0.003834
HALLMARK_MTORC1_SIGNALING	194	0.473788	1.766808	0.001678	0.007284	0.003834
HALLMARK_P53_PATHWAY	194	0.479256	1.787198	0.001678	0.007284	0.003834
HALLMARK_TNFA_SIGNALING_VIA_NFKB	197	0.528313	1.97611	0.001678	0.007284	0.003834
HALLMARK_ESTROGEN_RESPONSE_EARLY	192	0.488153	1.820779	0.001681	0.007284	0.003834
HALLMARK_HYPOXIA	190	0.528445	1.971652	0.001681	0.007284	0.003834
HALLMARK_E2F_TARGETS	189	0.637705	2.370316	0.001706	0.007284	0.003834
HALLMARK_G2M_CHECKPOINT	188	0.680848	2.528334	0.001706	0.007284	0.003834
HALLMARK_INTERFERON_ALPHA_RESPONSE	93	0.562764	1.918891	0.001748	0.007284	0.003834
HALLMARK_PANCREAS_BETA_CELLS	40	− 0.84889	− 2.53883	0.002132	0.008201	0.004316
HALLMARK_ALLOGRAFT_REJECTION	199	− 0.3919	− 1.50621	0.002469	0.008818	0.004641
HALLMARK_APICAL_JUNCTION	194	0.425713	1.58753	0.003356	0.010411	0.00548
HALLMARK_MYC_TARGETS_V1	192	0.43265	1.613757	0.003361	0.010411	0.00548
HALLMARK_APOPTOSIS	159	0.41949	1.510011	0.00354	0.010411	0.00548
HALLMARK_TGF_BETA_SIGNALING	54	0.51843	1.605989	0.005495	0.015263	0.008033
HALLMARK_MYC_TARGETS_V2	58	0.521398	1.636302	0.007449	0.019602	0.010317
HALLMARK_INFLAMMATORY_RESPONSE	197	0.37996	1.421208	0.008389	0.020849	0.010973
HALLMARK_UV_RESPONSE_DN	137	0.397836	1.40847	0.008757	0.020849	0.010973
HALLMARK_BILE_ACID_METABOLISM	112	− 0.40053	− 1.43725	0.011261	0.025594	0.01347
HALLMARK_ANDROGEN_RESPONSE	97	0.429447	1.468431	0.015762	0.034265	0.018034

### Correlation with tumor immune microenvironment

**Fig. 6:**
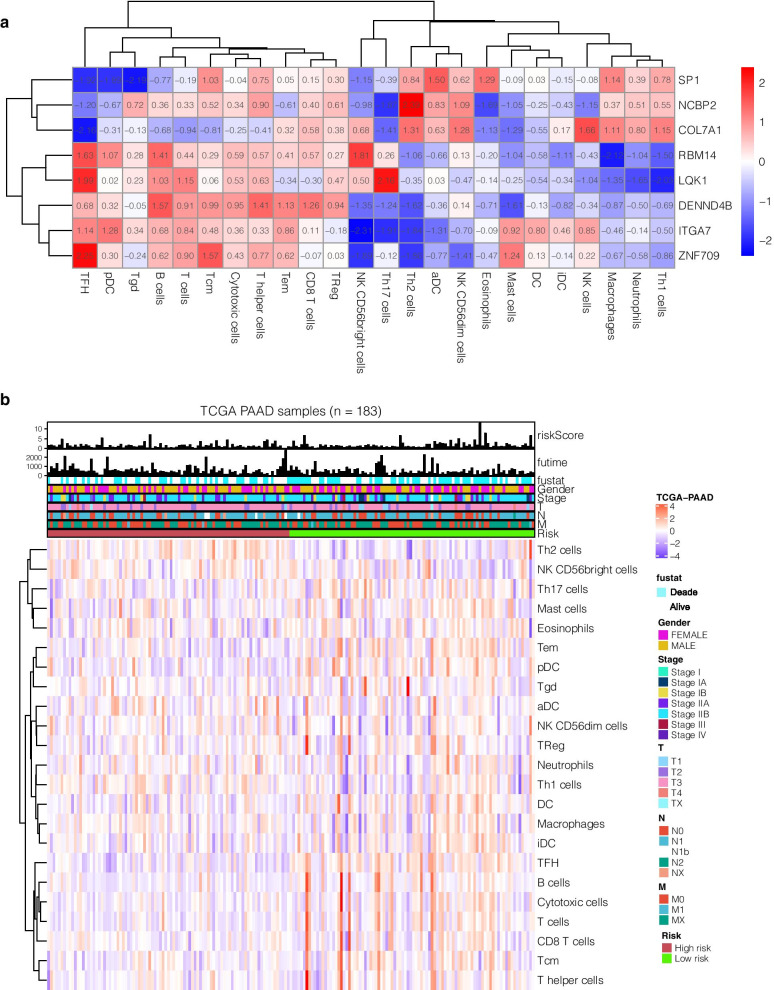
Correlation between prognostic markers and tumor immune microenvironment. **a** Correlations of 8 immune gene mRNAs and infiltration levels of 24 immune cells. **b** The heatmap shows the difference in infiltration levels of 24 immune cells in the high- and low-risk groups

**Fig. 7:**
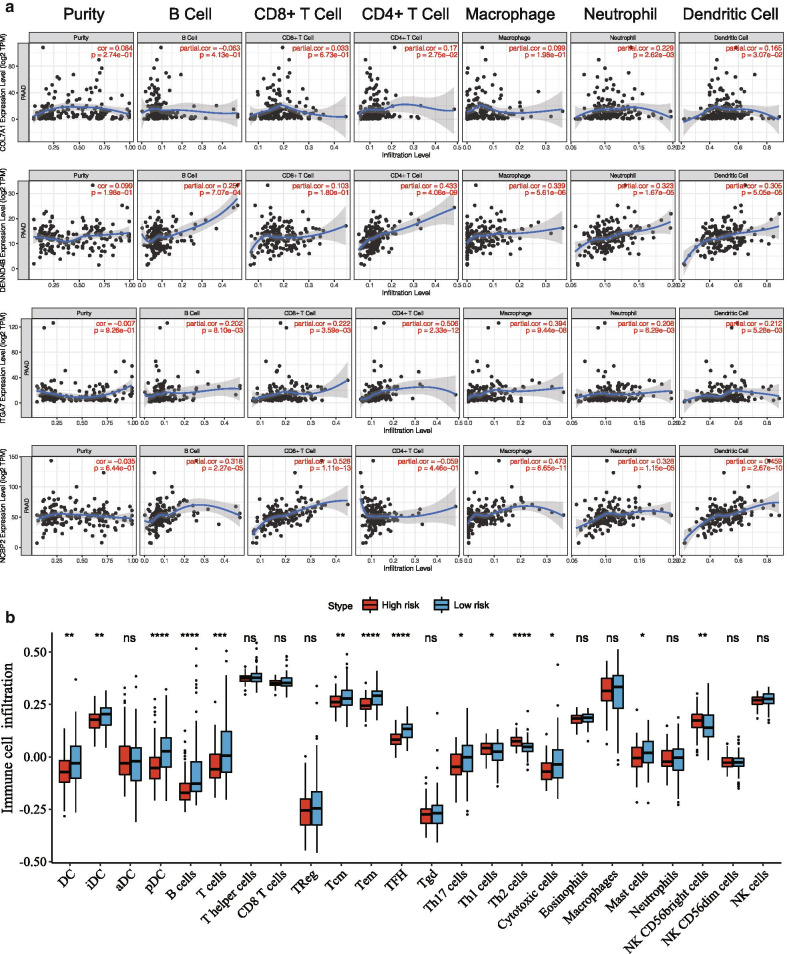
**a** The correlation between immune genes and immune cells was verified by using the TIMER database. Each point represents a sample, and the blue line represents the relationship between the expression level of each gene and the immune cell content. **b** Comparison of the difference in immune cell infiltration levels between high-risk group and low-risk group, ****P ≦ 1e−04, ***P ≦ 0.001, **P ≦ 0.01, *P ≦ 0.05, ns P ≧ 1

After quantifying 24 types of immune cells, Pearson test was used to calculate the correlation between risk genes and immune cells. DENND4B, ITGA7, and T cell lines such as T helper cells, Tcm, Tem, T cells, CD8 T cells, and TReg were highly positively correlated, whereas it was s negatively correlated with Th2 cells. RBM14 was negatively correlated with macrophages infiltration level, ZNF709 was significantly and positively correlated with TFH, and NCBP2 was positively correlated with Th2 cells (Fig. 6a).We observed that the infiltration levels of DC, iDC, pDC, B cells, T cells, Tcm, Tem, TFH, Th17 cells, and cytotoxic cells in the low-risk group were significantly higher than those in the high-risk group.Th1 cells and NK CD56dim cells in the high-risk group increased significantly compared with the low-risk group (Figs. 6b, 7b). Correlation analysis showed that DENND4B and ITGA7 were highly positively correlated with T helper cells, Tcm, Tem, T cells, CD8 T cells, and TReg. ZNF709 and SP1 were highly positively correlated with CD8 T cells, macrophage, and DC (Fig. 6a). TIMER database was used to further verify our results. DENND4B, ITGA7 and ZNF709, SP1 and CD4 T cells, Neutrophil, DC and other cells are highly positively correlated. ZNF709 and SP1 are positively correlated with CD8 T cells, Macrophage, DC and other cells (Fig. 7A).

## Discussion

Pancreatic cancer is one of the most malignant tumors of the digestive tract. The 5-year survival rate of patients with pancreatic cancer is less than 8%, and the average survival rate is less than 6 months. Considering that the mortality rate has been high in the recent decades, there is an urgent need to find effective biomarkers to promote and evaluate the diagnosis, treatment, and prognosis of pancreatic cancer [16–18]. The immune response in the microenvironment plays a decisive role in the progression, metastasis, and recurrence of tumors. Studies have confirmed that the low-immunogenic and immunosuppressive tumor microenvironment of pancreatic cancer is an important cause of poor prognosis. Following surgery, chemotherapy, and radiotherapy, immunotherapy has been considered as the fourth mode of cancer treatment. More and more clinical data show that cancer immunotherapy is a key step in clinical cancer treatment [19, 20]. Cancer management methods, especially non-small cell lung cancer, melanoma, urothelial cancer, and kidney cancer, and some preclinical and clinical trials have confirmed that immunotherapy has achieved encouraging results in many malignancies, including pancreatic cancer [21–23]. However, based on available data, it is clear that ICB has limited success in pancreatic cancer, which is also related to the low immunogenicity and immunosuppressive tumor microenvironment of pancreatic cancer [24, 25]. Based on the important role of immune response in pancreatic cancer, it is urgent to find new targets to provide powerful tumor prevention and control methods for regulating the body's immunity against tumors, and to add new content in the development of new adjuvant drugs targeted at tumor immunotherapy.

Gene markers, also known as classifiers, are often used to predict prognosis, sometimes even better than TNM staging methods, and have been reported in a variety of cancers [26–29]. Given the importance of immunity in pancreatic cancer, it is reasonable to speculate that immune-related genes have great promise in predicting prognosis. Multigene signals obtained from reliable algorithms will be superior to single molecules in predicting OS in pancreatic cancer. We constructed and validated a new immune-related gene signature that may be a potential target for cancer treatment, and they can improve the individualized prognosis of patients with pancreatic cancer.

We obtained 8 immune genes identified as independent prognostic factors for pancreatic cancer (ITGA7, COL7A1, SP1, NCBP2, RBM14, DENND4B, LQK1, ZNF709) through single factor COX regression, Lasso regression, and multifactor COX regression, which significantly affects the prognosis of patients with pancreatic cancer (Fig. 3). Some of them have been closely related to pancreatic cancer. The classic transcription factor SP1 has been confirmed to be closely related to the expression of multiple genes and the development of multiple cancers including PDAC. Target gene transcription can promote cell proliferation of pancreatic cancer cells [30, 31].

Previous literatures have also reported similar findings. For example, Wei-Dong Shi identified 12 human pancreatic cancer highly metastatic cell lines, SW19 × 90HM cells, including ITGA7 through microarray analysis [32]. Medicherla et al. showed that SD-208, a small molecule inhibitor of TGF-β receptor I kinase, downregulates the expression of the TGF-β regulatory gene COL7A1 and improves intervention in the development of pancreatic cancer [33]. However, NCBP2, RBM14, LQK1, and ZNF709 have not been reported to be related to pancreatic cancer. These may be the potential targets for the treatment of pancreatic cancer. We then used these genes to construct a clinical prediction model and calculate the risk score for each patient. Univariate COX regression analysis and multivariate COX regression analysis showed that the risk score was an independent predictor of prognosis. The Kaplan–Meier cumulative curve showed low risk. Patients with low risk scores survived significantly longer than patients with high risk scores (Fig. 1c). The highest area under the curve (AUC) values for this model were 0.755 (Fig. 1d). These results confirm the reliability of the model.

Pathway analysis found that high-risk groups were significantly positively associated with well-known cancer signaling pathways such as hypoxia, glycolysis, epithelial-mesenchymal transition, MTORC1 signaling pathway, P53 channel, apoptosis, E2F Targets, MYC Targets V1, and MYC Targets V2. Hypoxia is a common feature of malignant tumors, which is the result of increased oxygen demand due to cancer cell proliferation, tumor vascular dysfunction, and insufficient blood supply. Hypoxic tumor cells mainly rely on glycolysis to obtain energy and participate in the process of tumorigenesis, metastatic invasion, and treatment tolerance. This existing research confirms that hypoxia can regulate tumor immune microenvironment by regulating a variety of immune cells. Low oxygen levels significantly reduce the proliferation and activation of T lymphocytes, reduce the NKG2D receptors on NK cells and thereby inhibit the killing function of NK cells, increase tumor-associated macrophages to induce angiogenesis, and reduce inflammation to promote tumor progression [34–36]. On the other hand, it can affect tumor immune microenvironment through glycolysis pathway to affect immune cell infiltration and functional activation, and it is closely related to the efficacy of immunotherapy [37]. Growth factor signal transduction pathway mediated by mTOR complex 1 (mTORC1) promotes cancer metabolism through key enzymes that regulate metabolic pathways. Inhibition of mTOR C1 reduces glycolysis of cancer cells [38, 39].EMT can promote tumor cell infiltration and tumor metastasis and may also make tumor cells escape apoptosis induced by some factors [40, 41]. This process also plays a key role in regulating cellular plasticity in normal human tissues and tumor tissues. Many different cell subsets can be formed by EMT, that is, the heterogeneity of tumor cells is formed [42, 43]. P53 channel, E2F, and MYC are also common important signal pathways that affect tumor progression. This finding suggests that these immune risk genes are involved in the regulation of multiple signal pathways in pancreatic cancer and may have important biological and clinical significance.

New evidence confirms that disturbances in the immune response in the tumor microenvironment play a decisive role in tumor development [44].As a tumor killer, immune cells can interfere with molecular signals and play an important role in tumor biology such as tumor proliferation, metastasis, and invasion [45]. In our study, this signal was related to the immune response, immune microenvironment, and tumor purity of pancreatic cancer. By quantifying 24 types of immune cells, we observed that the low-risk group DC, iDC, pDC, B cells, T cells, Tcm, Tem, TFH, Th17 cells, and cytotoxic cells had a higher level of infiltration. Th1 cells and NK CD56dim cells in the high-risk group increased significantly (Fig. 7b). Correlation analysis showed that DENND4B and ITGA7 were highly positively correlated with T cell lines such as T helper cells, Tcm, Tem, T cells, CD8 T cells, and TReg. ZNF709 and SP1 are highly positively correlated with CD8 T cells, Macrophage, and DC. The accuracy of our results was verified using the TIMER database (Fig. 7a). The higher the level of these cells, the greater the benefit to the patient, and the better the efficacy of ICT. This further illustrates the importance of immune genes as prognostic markers in immune regulatory responses. The above indicates that our results may provide targets for immunotherapy.

## Conclusions

In conclusion, based on the TCGA-PAAD cohort, we identified immune-related markers with independent prognostic significance, verified, and analyzed their biological functions, to provide a feasible method to evaluate the prognosis of pancreatic cancer and provide valuable targets for immunotherapy of pancreatic cancer.

## Data Availability

All data used in this work can be acquired from the GDC portal (https://portal.gdc.cancer.gov/).

## Abbreviations

TCGA: The Cancer Genome Atlas
LASSO: The least absolute shrinkage and selection operator
KEGG: Kyoto encyclopedia of gene and genome
GO: Gene ontology
GSEA: Gene set enrichment analysis
PCA: Principal component analysis
ROC: The receiver operating characteristic
SsGSEA: The single-sample gene set enrichment analysis
TME: Tumor microenvironment
PCA: Principal component analysis

## References

[1] Wang K, Baldwin GS, Nikfarjam M, He H (2018). p21-activated kinase signalling in pancreatic cancer: New insights into tumour biology and immune modulation. Gastroenterol World J.

[2] Vennin C, Murphy KJ, Morton JP, Cox TR, Pajic M, Timpson P (2018). Reshaping the tumor stroma for treatment of pancreatic cancer. Gastroenterology.

[3] Garrido-Laguna I, Hidalgo M (2015). Pancreatic cancer: from state-of-the-art treatments to promising novel therapies. Nat Rev Clin Oncol.

[4] Delitto D, Wallet SM, Hughes SJ (2016). Targeting tumor tolerance: a new hope for pancreatic cancer therapy?. Pharmacol Ther.

[5] DeSelm CJ, Tano ZE, Varghese AM, Adusumilli PS (2017). CAR T-cell therapy for pancreatic cancer. J Surg Oncol.

[6] Akce M, Zaidi MY, Waller EK, El-Rayes BF, Lesinski GB (2018). The potential of CAR T cell therapy in pancreatic cancer. Front Immunol.

[7] Bharti G, Bhuwan G, Shrey M, Vrishketan S, Iris C, Oliver U, Yuguang B, Shweta L, Rajinder D (2018). NFκB in pancreatic stellate cells reduces infiltration of tumors by cytotoxic T cells and killing of cancer cells, via up-regulation of CXCL12. Gastroenterology.

[8] Riquelme E, Maitra A, McAllister FJ (2018). Immunotherapy for pancreatic cancer: more than just a gut feeling. Cancer Discov.

[9] Steele CW, Karim SA, Leach JD, Bailey P, Upstill-Goddard R, Rishi L, Foth M, Bryson S, Mcdaid K, Wilson Z (2016). CXCR2 inhibition profoundly suppresses metastases and augments immunotherapy in pancreatic ductal adenocarcinoma. Cancer Cells.

[10] Winograd R, Byrne KT, Evans RA, Odorizzi PM, Meyer AR, Bajor DL, Clendenin C, Stanger BZ, Furth EE, Wherry EJ (2015). Induction of T-cell immunity overcomes complete resistance to PD-1 and CTLA-4 blockade and improves survival in pancreatic carcinoma. Cancer Immunol Res.

[11] Jiang N, Qiao G, Wang X, Morse MA, Gwin WR, Zhou L, Song Y, Zhao Y, Chen F, Zhou X (2017). Dendritic cell/cytokine-induced killer cell immunotherapy combined with S-1 in patients with advanced pancreatic cancer: a prospective study. Clin Cancer Res.

[12] Yu GC, Wang LG, Han YY, He QY (2012). clusterProfiler: an R package for comparing biological themes among gene clusters. Omics.

[13] Bindea G, Mlecnik B, Tosolini M, Kirilovsky A, Waldner M, Obenauf AC, Angell H, Fredriksen T, Lafontaine L, Berger A (2013). Spatiotemporal dynamics of intratumoral immune cells reveal the immune landscape in human cancer. Immunity.

[14] Finotello F, Trajanoski Z (2018). Quantifying tumor-infiltrating immune cells from transcriptomics data. Cancer Immunol Immunother.

[15] Li B, Severson E, Pignon JC, Zhao H, Li T, Novak J, Jiang P, Shen H, Aster JC, Rodig S (2016). Comprehensive analyses of tumor immunity: implications for cancer immunotherapy. Genome Biol.

[16] Mork M, Quesada PR, Bannon S, Montiel MF, Fleming JB, Lynch PM, Bhutani MS, Lee JH, McAllister F (2019). Pancreatic cancer early detection and interception in an atypical case of Peutz–Jeghers syndrome. Pancreas.

[17] Crawford HC, Wallace MB, Storz P (2020). Early detection and imaging strategies to reveal and target developing pancreatic cancer. Expert Rev Anticancer Ther.

[18] Xu J, Liao K, Fu Z, Xiong Z (2019). A new method for early detection of pancreatic cancer biomarkers: detection of microRNAs by nanochannels. Artif Cells Nanomed Biotechnol.

[19] Fairfax BP, Taylor CA, Watson RA, Nassiri I, Danielli S, Fang H, Mahe EA, Cooper R, Woodcock V, Traill Z (2020). Peripheral CD8(+) T cell characteristics associated with durable responses to immune checkpoint blockade in patients with metastatic melanoma. Nat Med.

[20] Aslan K, Turco V, Blobner J, Sonner JK, Liuzzi AR, Nunez NG, De Feo D, Kickingereder P, Fischer M, Green E (2020). Heterogeneity of response to immune checkpoint blockade in hypermutated experimental gliomas. Nat Commun.

[21] Xie C, Duffy A, Brar G, Fioravanti S, Mabry-Hrones D, Walker M, Monge Bonilla C, Wood BJ, Citrin DE, GilRamirez EM (2020). Immune checkpoint blockade in combination with stereotactic body radiotherapy in patients with metastatic pancreatic ductal adenocarcinoma. Clin Cancer Res.

[22] Wessely A, Steeb T, Erdmann M, Heinzerling L, Vera J, Schlaak M, Berking C, Heppt MV (2020). The role of immune checkpoint blockade in uveal melanoma. Int J Mol Sci.

[23] Snook JP, Soedel AJ, Ekiz HA, O'Connell RM, Williams MA (2020). Inhibition of SHP-1 expands the repertoire of antitumor T cells available to respond to immune checkpoint blockade. Cancer Immunol Res.

[24] Zhao J, Wen X, Tian L, Li T, Xu C, Wen X, Melancon MP, Gupta S, Shen B, Peng W (2019). Irreversible electroporation reverses resistance to immune checkpoint blockade in pancreatic cancer. Nat Commun.

[25] Zhang Q, Green MD, Lang X, Lazarus J, Parsels JD, Wei S, Parsels LA, Shi J, Ramnath N, Wahl DR (2019). Inhibition of ATM increases interferon signaling and sensitizes pancreatic cancer to immune checkpoint blockade therapy. Cancer Res.

[26] Liu Y, Wu L, Ao H, Zhao M, Leng X, Liu M, Ma J, Zhu J (2019). Prognostic implications of autophagy-associated gene signatures in non-small cell lung cancer. Aging (Albany NY).

[27] Bao ZS, Li MY, Wang JY, Zhang CB, Wang HJ, Yan W, Liu YW, Zhang W, Chen L, Jiang T (2014). Prognostic value of a nine-gene signature in glioma patients based on mRNA expression profiling. CNS Neurosci Ther.

[28] Cheng W, Ren X, Zhang C, Cai J, Liu Y, Han S, Wu A (2016). Bioinformatic profiling identifies an immune-related risk signature for glioblastoma. Neurology.

[29] Wei C, Liang Q, Li X, Li H, Liu Y, Huang X, Chen X, Guo Y, Li J (2019). Bioinformatics profiling utilized a nine immune-related long noncoding RNA signature as a prognostic target for pancreatic cancer. J Cell Biochem.

[30] Zhang B, Song L, Cai J, Li L, Xu H, Li M, Wang J, Shi M, Chen H, Jia H (2019). The LIM protein Ajuba/SP1 complex forms a feed forward loop to induce SP1 target genes and promote pancreatic cancer cell proliferation. J Exp Clin Cancer Res.

[31] Qian Y, Yao W, Yang T, Yang Y, Liu Y, Shen Q, Zhang J, Qi W, Wang J (2017). aPKC-iota/P-Sp1/Snail signaling induces epithelial–mesenchymal transition and immunosuppression in cholangiocarcinoma. Hepatology.

[32] Shi WD, Meng Z-Q, Chen Z, Lin J-H, Zhou Z-H, Liu L-M (2009). Identification of liver metastasis-related genes in a novel human pancreatic carcinoma cell model by microarray analysis. Cancer Lett.

[33] Medicherla S, Li L, Jing YM, Kapoun AM (2007). Antitumor activity of TGF-β, Inhibitor is Dependent on the Microenvironment. Anticancer Res.

[34] Marques MW, Lima NB, Michereff SJ, Camara MPS, Souza CRB (2012). First report of mango dieback caused by Pseudofusicoccum stromaticum in Brazil. Plant Dis.

[35] Tian L, Goldstein A, Wang H, Ching Lo H, Sun Kim I, Welte T, Sheng K, Dobrolecki LE, Zhang X, Putluri N (2017). Mutual regulation of tumour vessel normalization and immunostimulatory reprogramming. Nature.

[36] Jiao S, Subudhi SK, Aparicio A, Ge Z, Guan B, Miura Y, Sharma P (2019). Differences in tumor microenvironment dictate T helper lineage polarization and response to immune checkpoint therapy. Cell.

[37] Leone RD, Zhao L, Englert JM, Sun IM, Oh MH, Sun IH, Arwood ML, Bettencourt IA, Patel CH, Wen J (2019). Glutamine blockade induces divergent metabolic programs to overcome tumor immune evasion. Science.

[38] Zhao X, Jiang P, Deng X, Li Z, Tian F, Guo F, Li X, Wang S (2016). Inhibition of mTORC1 signaling sensitizes hepatocellular carcinoma cells to glycolytic stress. Am J Cancer Res.

[39] Chen X, Zhu Y, Wang Z, Zhu H, Pan Q, Su S, Dong Y, Li L, Zhang H, Wu L (2016). mTORC1 alters the expression of glycolytic genes by regulating KPNA2 abundances. J Proteomics.

[40] Zhao XH, Wang ZR, Chen CL, Di L, Bi ZF, Li ZH, Liu YM (2019). Molecular detection of epithelial-mesenchymal transition markers in circulating tumor cells from pancreatic cancer patients: Potential role in clinical practice. World J Gastroenterol.

[41] Sato M, Matsumoto M, Saiki Y, Alam M, Nishizawa H, Rokugo M, Brydun A, Yamada S, Kaneko MK, Funayama R (2020). BACH1 promotes pancreatic cancer metastasis by repressing epithelial genes and enhancing epithelial-mesenchymal transition. Cancer Res.

[42] Wu X, Liu Z, Guo K, Ma G, Song S (2019). Inactivation of ATF-2 enhances epithelial-mesenchymal transition and gemcitabine sensitivity in human pancreatic cancer cells. J Cell Biochem.

[43] Wei R, Penso NEC, Hackman RM, Wang Y, Mackenzie GG (2019). Epigallocatechin-3-gallate (EGCG) suppresses pancreatic cancer cell growth, invasion, and migration partly through the inhibition of Akt pathway and epithelial-mesenchymal transition: enhanced efficacy when combined with gemcitabine. Nutrients.

[44] Tron L, Belot A, Fauvernier M, Remontet L, Bossard N, Launay L, Bryere J, Monnereau A, Dejardin O, Launoy G (2019). Socioeconomic environment and disparities in cancer survival for 19 solid tumor sites: An analysis of the French Network of Cancer Registries (FRANCIM) data. Int J Cancer.

[45] Arnoletti JP, Zhu X, Almodovar AJ, Veldhuis PP, Sause R, Griffith E, Corpus G, Chang JC, Fanaian N, Litherland SA (2017). Portal venous blood circulation supports immunosuppressive environment and pancreatic cancer circulating tumor cell activation. Pancreas.

